# Brain structures and their association with executive and attentional abilities in very preterm 8-year-old children

**DOI:** 10.1007/s00429-025-03047-8

**Published:** 2025-11-22

**Authors:** Marion Décaillet, Yasser Alemán-Gómez, Mikkel Schöttner Sieler, Solange Denervaud, Cléo Huguenin-Virchaux, Laureline Besuchet, Céline J. Fischer Fumeaux, Patric Hagmann, Juliane Schneider

**Affiliations:** 1 Department of Radiology, Lausanne University Hospital and University of Lausanne, Lausanne, Switzerland; 2 The Sense Innovation and Research Center, Lausanne and Sion, Switzerland; 3 Clinic of Neonatology, Department of Woman-Mother-Child, Lausanne University Hospital and University of Lausanne, Lausanne, Switzerland; 4 CIBM Center for Biomedical Imaging, Lausanne, Switzerland; 5 MRI Animal imaging and technology, Polytechnical School of Lausanne, Swiss Federal Institute of Technology Lausanne (EPFL), Lausanne, Switzerland

**Keywords:** Very preterm birth, Cortical thickness, Executive function, Attention, Morphometry, Connectivity

## Abstract

Very preterm (VPT) children are prone to a variety of neurodevelopmental impairments, particularly regarding their attention and executive functions (i.e., inhibition, shifting, and working memory). Here, we aimed to investigate whether morphometric and connectivity characteristics from key brain regions associated with attention and executive functions may underlie their difficulties. Thirty-three VPT children (M_gestational age_ = 27.22 weeks, SD = 1.36) aged 8–10 years (M_age_ = 8.85, SD = 0.49, 17 girls) underwent a brain magnetic resonance imaging (MRI) session alongside neurodevelopmental testing. We performed a factor analysis to group the different behavioural variables measuring executive and attentional capacities. The analysis yielded a tripartite structure wherein the first factor was predominantly characterized by inhibitory abilities, the second by attentiveness, and the third by flexibility. To encompass brain regions involved in attention and executive processes, based on functional MRI meta-analyses, we selected the anterior cingulate (ACC) and the dorsolateral prefrontal cortices (DLPFC). From T1-weighted and diffusion MRI images we estimated their cortical thickness, fractional anisotropy, volume, cortical surface area, and betweenness centrality. Significant negative associations were observed for cortical thickness after multiple comparison corrections and adjustments for age and sex. Thinner cortex was related to higher inhibitory, flexibility, and attentional functioning. While these associations were independent of the hemispheres, the association with the inhibitory abilities was stronger in the DLPFC than in the ACC. No associations were found for the other brain measures. These findings provide new insights into brain structures underpinning executive and attentional abilities in VPT children at school age.

**Supplementary Information:**

The online version contains supplementary material available at 10.1007/s00429-025-03047-8.

## Introduction

Very preterm birth (i.e., < 32 gestational weeks) has been demonstrated to result in a range of cerebral impairments and brain dysmaturation (Inder et al. 2023). Despite advances in medical care and a decrease in mortality and severe disabilities, very preterm children continue to exhibit an elevated risk of various adverse long-term neurodevelopmental outcomes (Anderson 2014; Doyle et al. 2021; Marlow et al. 2021). While these impairments may concern a wide range of cognitive domains, higher-order cognitive control such as executive functions and attentional abilities are commonly reported as altered in very preterm children (Aarnoudse-Moens et al. 2009; Inder et al. 2023; Mulder et al. 2009). These functions encompass the core capacities for working memory, inhibition and shifting, allowing children to generate goal-directed behaviours (Baggetta and Alexander 2016). Moreover, these functions influence higher-order cognitive skills and school readiness (Rose et al. 2011), which renders them essential for daily life. Only a few studies have investigated the brain-function relationship in very preterm children at school age (e.g., Kallankari et al. 2021; Mürner-Lavanchy et al. 2018; Réveillon et al. 2020).Yet, a comprehensive characterization of the neural specificities underlying each core executive and attentional skills would facilitate a more profound understanding of their difficulties to ultimately provide more adapted care.

Quantitative measures of brain anatomy, encompassing morphometric and diffusion-derived measures, such as cortical thickness, surface area, volume, and fractional anisotropy (FA), provide detailed information about structural, microstructural and connectivity properties of the brain. First, cortical thickness represents the distance between the innermost and outermost edges of the cerebral cortical grey matter (i.e., the brain’s outer layer) (Gale and Huff 2017). Despite the lack of consensus regarding the timing of developmental trajectory change (Walhovd et al. 2016), research has demonstrated that cortical thickness undergoes an initial thickening phase during the early years, followed by a subsequent thinning phase (Ducharme et al. 2016), with different rhythms for different brain regions (Sakai 2020; Tau and Peterson 2010). This refinement of synaptic connections ultimately leads to better cognition associated with a thinner cortex (Giedd et al. 1999). It is noteworthy that while most of the regions are thinning after five years, the left anterior and posterior perisylvian regions, known to be involved in language processing (Brauer et al. 2008), are still thickening during late childhood (Sowell et al. 2004). Second, the number of cortical columns characterizes cortical surface area (Panizzon et al. 2009), that undergoes the same type of developmental trajectories as cortical thickness. However, while cortical surface peaks around the age of nine, followed by a subsequent reduction (Wierenga et al. 2014), the trajectory varies largely between brain regions, with frontal areas developing at the last stage (Ducharme et al. 2015). Third, cortical volume is defined as the combination of cortical thickness and surface area (Panizzon et al. 2009). Consequently, its development is contingent on the specific development of brain areas according to their cortical thickness and cortical surface area development. While most of the cortical regions follow a linear decline, no changes occur in the bilateral medial temporal areas, temporal poles, and the anterior cingulate cortex (ACC; Ducharme et al. 2015). Finally, FA has been shown to increase with age in many white matter tracts, reflecting higher neural organization (Qiu et al. 2008; Schmithorst et al. 2002). Beyond local microstructural properties, metrics of brain connectivity have become essential for characterizing neural networks. Notably, betweenness centrality quantifies a region’s importance in facilitating information transfer across the brain network (Liu et al. 2017). This metric develops early, with a significant increase in the first year of life, after which it stabilizes (Bagonis et al. 2023; Hagmann et al. 2010).

Several studies have characterized the brain of preterm infants from birth to adulthood. Common brain injuries following very preterm birth encompass three different types, white-matter injury, germinal matrix-intraventricular haemorrhage, and cerebellar haemorrhage (Inder et al. 2023). In addition, besides overt brain lesions, both the grey and the white matter undergo a certain form of dysmaturation of various degrees (Inder et al. 2023). In recent years, while dysmaturation is commonly reported, severe lesions are decreasing, and there are more subtle damages. These latter can be associated with altered brain functional and structural connectivity such as decreased functional network connectivity and disruptions in both short- and long-distance structural connections (for a review, see Inder et al. (2023) or Smyser et al. (2019). Long-lasting brain alterations are still found in very preterm children at school age and adolescence and affect most of the brain metrics. Firstly, very preterm children present reduced cortical volumes, especially in temporal regions, compared to full-term children (Kelly et al. 2024; Zhang et al. 2015), with a slower increase during childhood (Matthews et al. 2018; Ment et al. 2009; Thompson et al. 2020). Depending on the region, mixed results regarding cortical thickness were found. Very preterm children showed thicker cortex in widespread regions (De Gamarra-Oca et al. 2024; Nam et al. 2015), alongside a delayed developmental trajectory (Vandewouw et al. 2020). While cortical thinning seems to occur before the age of seven in full-term children, this thinning continues at least until the age of twelve in very preterm children (Mürner-Lavanchy et al. 2014) and occurs with more significant decrease (Vandewouw et al. 2020). However, smaller regions, such as the right ACC, right superior temporal sulcus, and right anterior insula, demonstrate thinner cortical thickness in very preterm children, as well as bilateral parahippocampal regions, left insula, right temporoparietal junction and the posterior part of the right inferior frontal sulcus in adolescents and adults compared to full-term peers (Lax et al. 2013; Nam et al. 2015). Very preterm children exhibit reduced FA in many white matter tracts (Young et al. 2018; Zhou et al. 2024). In addition, smaller surface area and gyrification index are found in very preterm children at school age (Mürner-Lavanchy et al. 2018; Zhang et al. 2015). Finally, few studies have investigated the betweenness centrality in preterm infants, and mixed results have been found. While (Gozdas et al. 2018) found no significant difference between very preterm and full-term infants, an increased leftward asymmetry was found in the fronto-limbic system (i.e., medial fronto-orbital gyrus, superior temporal gyrus, and hippocampus) in preterm infants (Lee et al. 2021). In addition, Argyropoulou et al. (2022) showed a decreased betweenness centrality in the left frontoparietal operculum in preterm infants with low‑grade intraventricular haemorrhage compared to preterm infants without brain damage. However, it seems that this has not been assessed at a later age. Therefore, as very preterm children present varying long-lasting brain alterations, it is essential to understand their impact on cognition.

Few studies have investigated the relation between brain structures and cognition in very preterm children and young adults, especially regarding executive function and attention capacities, with conflicting results depending on which brain regions and cognitive functions were examined. First, in very preterm children thicker cortical thickness was associated with better executive functions in the left superior temporal gyrus and sulcus, the lateral occipital cortex, and the right middle temporal gyrus (Mürner-Lavanchy et al. 2018), regions involved in attention (Murray and Wojciulik 2004) and perception (Grill-Spector et al. 2001; Volfart et al. 2023). Complementary, while full-term children showed negative correlations between working memory and cortical thickness of the right inferior parietal, a region involved in perception and attention (Baldo and Dronkers 2006; Vandenberghe et al. 2012), and of the left precuneus (Mürner-Lavanchy et al. 2018), a region important for attention and memory (Cavanna and Trimble 2006), their executive control was positively associated with the superior parietal cortical thickness, a region normally involved in sensory perception and working memory (Koenigs et al. 2009) but negatively associated with the right parahippocampal gyrus (Li et al. 2020), which is important for encoding and maintaining associative information (Luck et al. 2010). Moreover, Mürner-Lavanchy et al. (2018) showed in very preterm children a negative association between cortical surface area and working memory in the right lateral occipital gyrus, a region associated with attention and perception (Murray and Wojciulik 2004; Volfart et al. 2023), and cognitive motor speed in the left inferior temporal gyrus, which has been shown to be important for perception visual and language comprehension and emotion regulation (Lin et al. 2020). In addition, while several studies (De Gamarra-Oca et al. 2024; Murray et al. 2016; Vollmer et al. 2017) found no significant relation between volume and cognitive measures in very-preterm children and adolescents, Li et al. (2020) showed a positive association between cortical volume and attention in the left medial orbitofrontal cortex, which is known to be involved in reward processing and decision-making (Rolls and Grabenhorst 2008; Zhang et al. 2022). In full-term children, they also reported a negative association between working memory and the left medial orbital frontal cortex and left pars opercularis gyrus volumes, a region associated with language processes (Foundas et al. 1998). Moreover, while very preterm children demonstrated positive associations between attention and FA in the left cingulum and superior longitudinal fasciculus (Murray et al. 2016), Kennedy et al. (2021) showed that very preterm children with neurodevelopmental impairment had a lower FA in the superior longitudinal fasciculus, uncinate fasciculus, and right hemisphere corticospinal tract, various tracts which are mostly involved in attention, working memory, language, and motor processes (Bettcher et al. 2016; Natali et al. 2025; Ribeiro et al. 2023; Von Heide et al. 2013). Finally, to our knowledge, no studies have investigated the relationship between cognition and betweenness centrality in very preterm children. Altogether, these studies revealed mixed associations between different brain areas and metrics, and executive functions and attentional abilities, suggesting that the nature of these relationships varies based on the functions examined and the regions implicated, even when those regions pertain to the same skills.

While these limited studies provide useful insights in the brain-function relationships with various associations, most of them did not focus on specific brain regions associated with certain cognitive functions but performed whole-brain analyses. Consequently, previously identified regions were predominantly engaged in perception, attention, and memory, and yet the implications of areas recognized as critical for executive functions remain uncertain. The dorsolateral prefrontal cortex (DLPFC) and the ACC and their functional connectivity have often been reported to be involved in executive functions and attention, in a distinct manner (Engelhardt et al. 2019; Johnston et al. 2007; Panikratova et al. 2020). While the DLPFC is more involved in the different executive functions (Panikratova et al. 2020), especially inhibitory control (Angius et al. 2019; Sandrini et al. 2020), the ACC contributes to attention (Wu et al. 2017), conflict monitoring and cognitive control (Kerns et al. 2004). Moreover, abnormal activity has been found in these two regions in ADHD patients (Hart et al. 2013; Zarka et al. 2021). In addition, the DLPFC and ACC are especially engaged for high demanding tasks (Engelhardt et al. 2019), which are common in neuropsychological assessments, and when very preterm children present difficulties (Décaillet et al. 2023). Therefore, the present study focused on these two regions to encompass the different types of executive functions and attentional processes. Previous studies showed both morphometric and connectivity implications, consequently we sought to thoroughly characterize the DLPFC and the ACC by extracting five brain measures, the cortical thickness, the cortical surface area, the cortical volume, the FA, and the betweenness centrality. As a result, this study was aimed to study the relationship between the DLPFC and the ACC and the executive functions and attentional capacities of very preterm school-aged children. We hypothesized that enhanced maturation, as measured by brain metrics at age eight, would be associated with cognitive test performance evaluating attention and executive functions. In view of the absence of consensus, no assumptions were made regarding the directionality of these associations.

## Method

### Participants

Fifty-one very preterm neonates were originally enrolled in the main project *Long-term impact of early nutritional and pain management in very preterm infants on brain health and function* between February 2011 and May 2013. This was a longitudinal study that followed the neonates from birth to eight years of age, with the present study taking place during the final follow-up phase from October 2019 to October 2021. For the present sub-study, eleven children were not included/recruited because of death (*n* = 2), lost contact at a previous follow-up visit (*n* = 2), complex social situation (*n* = 2), unreachable parents (*n* = 2), refusal to participate (*n* = 2), or incomplete testing (*n* = 1). Additionally, five children did not undergo MRI, and two had to be excluded due to excessive movement. As a result, thirty-three children were included, aged eight to ten years chronological (M_age_ = 8.85, SD = 0.49, 17 girls). Each parent signed a written consent for their child, and verbal assent was provided by each child. The only exclusion criterion at this stage was parental refusal to be informed of incidental findings on MRI, and none were excluded.

### Socioeconomic status

The parental socioeconomic status (SES) was evaluated using the Largo scale (Largo et al. 1989), with a score of 1 indicating the highest level and a score of 6 the lowest.

### Neuropsychological tests

During the follow-up appointment at eight years, children performed various tests. We selected five of them to encompass the different types of executive functions and attentional capacities, as described below. It took place in a quiet room in the Development Unit of the Lausanne University Hospital. Psychologists and neuroscience researchers assessed the different tests.

#### Wechsler intelligence scale for children 5th edition (WISC-V)

Through a range of various subtests, this battery evaluates the intelligence of children (Wechsler 2016). In the present study, we selected the *Working Memory Index*, which is subdivided into two subtests. The first is the *Picture Span* subtest, in which children are shown a sequence of pictures and then instructed to select them in sequential order from multiple pictures. Second, the *Digit Span* subtest asks children to recall a sequence of numbers in original, reverse, and ascending order.

#### Nepsy – II

This battery (Korkman et al. 2008) evaluates various neuropsychological abilities of children through 32 subtests. For the current study, we retained solely the *Design Fluency* subtest, which is a measure of executive functioning. It assesses cognitive flexibility, working memory, and initiation capacity. Children were instructed to create as many distinct designs as possible by connecting five dots within 60 s, once with structured positioning and again with random positioning. The number of designs produced for the two subtests (i.e., structured and random) were summed and converted into a standard score.

#### Conners continuous performance test 3rd edition (CPT3)

This computerized test assesses attentional capacities (Conners 2014). In this task, letters scroll across the screen, and the children are asked to press the spacebar as soon as a letter is presented, except for the letter “X.” It is composed of 360 trials and lasts 14 min. Seven different attentional scores are computed. For the present study, we only selected two measures: the *omission error*, which is the failure rate to respond to the target and evaluates attentiveness, and the *commission error*, which is computed as the response rate for the non-targets and represents the inhibition capacity. Both scores were transformed into T-scores as the population mean is not known.

#### Flanker task

This computerized test evaluates different executive function measures. We used a child fish version of the task where fish replace arrows (Schonert-Reichl et al. 2015). Fish are displayed in a line, and children are asked to feed the target fish by inhibiting distractors by pressing a key on the same side from which the fish is looking. The task consists of three blocks with different rules. In the first one, blue fish are displayed, and children are instructed to feed the middle one; then in the second block, the fish turn pink, and the target fish are the four other ones, and in the third block, both rules are mixed, and target fish are determined by their colour. The first two blocks are composed of 17 trials, and the third block comprises 65. The task was displayed and controlled using Presentation^®^ software (Version 23.0, Neurobehavioral Systems, Inc., Berkeley, CA, www.neurobs.com). For the current study, we selected two measures: the *inhibition* measure, which was computed as the accuracy of the second block and evaluates the capacity to inhibit the former rule, and the *global executive functions* measure, which was computed as the accuracy of the third block and represents the ability to recruit the three core executive functions, particularly shifting.

### MRI data acquisition

Anatomical MRI data were acquired using a 3.0T Siemens Prisma scanner equipped with a 64-channel head coil at the CIBM Centre for Biomedical Imaging, Lausanne University Hospital (CIBM-CHUV). Each child completed an MRI session following a study-specific protocol designed for this study that included three acquisition sequences.

First, high-resolution structural images were obtained using a 3D T1-weighted Magnetization Prepared Rapid Gradient Echo (MPRAGE) sequence (acquisition time: 5 min) with the following parameters: repetition time (TR) = 2000 ms, echo time (TE) = 2.47 ms, inversion time (TI) = 900 ms, field of view (FOV) = 208 × 256 × 256 mm³, flip angle = 8°, and isotropic voxel size = 1 × 1 × 1 mm³.

Second, diffusion-weighted images were acquired using a High Angular Resolution Diffusion Imaging (HARDI) protocol (acquisition time: 13 min). Parameters were: TR = 5100 ms, TE = 80 ms, FOV = 233.6 × 233.6 × 266.6 mm³, flip angle = 90°, voxel size = 1.6 × 1.6 × 3.1 mm³, with parallel imaging acceleration factors of PE = 3 and slice = 2. Diffusion encoding was performed across four shells with b-values of 700, 1000, 2000, and 3000 s/mm². A total of 137 diffusion-weighted directions were sampled, along with one b = 0 image.

During anatomical data acquisition, children watched a movie of their choice to help them remain still and comfortable. Noise was attenuated using headphones, and additional foam padding was placed around the head to minimize motion. No sedation or contrast agent was used. Functional images were also acquired as part of the session; however, these data are not analysed in the present study. The entire scanning session lasted approximately 60 min.

### Processing MRI data

MRI images were converted and organized according to the BIDS standards. The images corresponding to each individual subject were processed using a combination of different image processing suites such as FreeSurfer (v7.2.1, Fischl 2012), FSL (v6.0.4, Jenkinson et al. 2012), MRtrix3(v3.0.2, Tournier et al. 2019), and ANTs (v2.5.0, Avants et al. 2011) within a containerized computational environment.

### Structural T1-weighted imaging processing

T1-weighted images were processed using FreeSurfer to extract cortical surfaces (pial and white), cortical maps (e.g., thickness and curvature), and a cortical parcellation based on the Desikan-Killiany atlas, which defines 34 regions per hemisphere.

This parcellation serves as the first level (scale 1) in the multiscale cortical parcellation framework developed by Cammoun et al. (2012), which progressively subdivides the cortex into finer regions. The framework consists of five hierarchical scales, with the final parcellations comprising 68, 114, 216, 446, and 1002 regions, respectively. These parcellations were mapped onto individual subject spaces, enabling both surface-based cortical segmentations and corresponding volumetric parcellations for structural and connectivity analyses. Additionally, to generate a volumetric parcellation of the white matter adjacent to the cortex, these parcellations were projected 2 mm into the gyral white matter.

### Cortical region of interest segmentation using neurosynth

To define this study’s Regions of Interest (ROIs), we utilized Neurosynth (Yarkoni et al. 2011), a large-scale meta-analytic tool that generates probabilistic activation maps based on keyword searches. Specifically, we searched for the terms *Anterior Cingulate* and *Working memory: association test* (i.e., for the DLPFC), which yielded an activation map in MNI (Montreal Neurological Institute) space corresponding to regions associated with these terms. This map was then intersected with the finest-resolution cortical parcellation (scale 5). To determine the final bilateral ROIs for each hemisphere, we identified the parcels whose surface area overlapped most extensively with the Neurosynth-derived activation map. These selected parcels were then grouped to form two distinct ROIs: the ACC and the DLPFC.

The volume, surface area, and mean cortical thickness were computed for each region of the multi-scale cortical parcellation and the grouped regions of interest.

### Diffusion-weighted imaging processing

Individual diffusion MR (dMRI) images underwent a series of correction steps using Mrtrix3 and FSL. These steps included denoising, bias correction, distortion correction (Andersson et al. 2003), motion correction, and eddy current correction (Andersson and Sotiropoulos 2016). Brain extraction was performed using SynthStrip (v1.6.0), ensuring accurate tissue segmentation.

Next, the diffusion tensor and its derived scalar maps, such as FA, were estimated using MRtrix3. To estimate the fiber intravoxel orientation function (fODs) estimation, multi-shell multi-tissue constrained spherical deconvolution (MSMT-CSD) was performed using *dwi2fod*, generating white matter fiber orientation distribution functions. Response functions were computed using the Dhollander algorithm (Dhollander et al. 2018). Deterministic streamline tractography was performed using MRtrix3’s *tckgen* with the SDSTREAM algorithm, incorporating ACT priors, a step size of 0.5 mm, a maximum length of 250 mm, and a minimum length of 5 mm. A total of 10,000,000 streamlines were generated per subject, and the connectivity matrix was obtained for each cortical parcellation.

The tractogram was filtered using SIFT2 (Spherical-deconvolution Informed Filtering of Tractograms; Smith et al. 2015) to improve the quantitative accuracy of streamline density in relation to underlying white matter structure. The resulting weighted streamlines were then used to compute the structural connectivity matrix by mapping them onto the multi-scale cortical parcellation. Specifically, for each scale, streamlines were assigned to pairs of regions based on their endpoints, and connection weights were derived using the SIFT2-derived streamline weights.

The mean FA values were computed in the segmented white matter located close to each cortical region and both regions of interest (DLPFC and ACC). From the multi-scale connectivity matrices, we extracted the betweenness centrality.

### Factor analysis

Due to the large number of neuropsychological scores compared to the number of children, we used exploratory factor analysis for dimensionality reduction using a similar procedure as Schöttner et al. (2023).

#### Pre-processing

First, CPT-3 scores were inverted compared with the other tests, and new scores were computed by subtracting the score from the maximum score. This way, the interpretation of all variables was the same in that higher values indicate a better performance. Missing values (for more details, see Table 2) were imputed using the Multiple Imputation by Chained Equations algorithm (MICE, Van Buuren and Groothuis-Oudshoorn 2011). Finally, all scores were transformed into z-scores.

#### Exploratory factor analysis (EFA)

We performed exploratory factor analysis using the package “Factor Analyzer” (v0.4.0., EducationalTestinService, 2021) implemented in Python. We used maximum likelihood factor analysis and Promax rotation, meaning that our factors can be correlated. Parallel analysis (Horn 1965) was employed to find the optimal number of factors. It indicated that the two- and three-factor solutions were the best choices, and the additional variance explained by the factors offered only incremental improvements. The three-factor model was selected as the three factors were distributed in a manner that was more clinically coherent. Factor 1 was primarily driven by inhibitory abilities, Factor 2 by attentiveness, and Factor 3 by flexibility (see *Supplementary Materials* for the factor loading matrix), and will therefore be referred to as the Inhibition Factor, Attentiveness Factor and Flexibility Factor, respectively. The factor scores for the three factors were then extracted for this model using Bartlett’s method (Bartlett 1937).

### Data analysis

We tested the normality with Shapiro-Wilk test, histograms, and boxplots. We performed partial correlations adjusted for age, sex and the SES between the three factors and the five brain measures (i.e., cortical thickness, cortical surface area, cortical volume, FA, and betweenness centrality). Pearson correlations were used when variables were normally distributed, and Spearman correlations otherwise. The False Discovery Rate method was applied to correct for multiple comparisons.

Then, based on these correlations, linear regressions were computed to investigate how the cortical thickness of the ACC and the DLPFC were related to the three factors. As the three factors had some outliers, the function winsorize() from the *datawizard* package was used. In addition, the associations between the factors, cortical thickness, and age, sex and SES were tested for inclusion in the models. Sex was only associated with Inhibition Factor and Attentiveness Factor, and age and SES were not correlated with any of the factors or cortical thickness (for details see *Supplementary Materials*). Therefore, age and SES were not included in the models, and while models for Inhibition Factor and Attentiveness Factor were computed as “winsorize (Factor, threshold = 0.2) ~ sex + thickness + thickness: hemisphere + thickness: region + thickness: hemisphere: region”, models were constructed without sex for Flexibility Factor. We compared the full model with interactions with the null model (Factor ~ 1) to avoid Type I errors using the function anova() with the “Chisq” test. Predictors were tested for collinearity with the function vif() from the *car* package.

Finally, in order to ascertain whether the relationship between the cortical thickness and the executive and attentional functioning was specific to the DLPFC and the ACC, or whether it was merely a reflection of a general relationship between the cortical thickness and the cognition, we selected regions which are not related to the executive functions and attentional functioning (i.e., superior temporal, inferior temporal, fusiform, lateral occipital, and superior parietal cortices) as well as the total brain cortical thickness. We computed linear regressions for these brain regions to evaluate if their cortical thickness was related to the three factors. As in the previous regressions, we first tested whether sex, age and SES were associated with the factors and the cortical thickness, and only predictors with significant associations were included in the models. The full models were also compared with the null models.

Analyses were done with Jamovi (version 2.4, The jamovi project 2023) Computer Software and R (version 4.4.0, R Core Team 2022).

## Results

### Demographics

The very preterm children presented various complications during the neonatal period and difficulties at school age (for details see Table 1).

**Table 1:** Neonatal and school-age characteristics of the very preterm cohort

Demographics		M (s.d.)[range]/*n* (%)
N (%girls)		33 (51%)
Age in years, M (s.d.)		8.85(0.49) [8.0–10.0]
Socioeconomic status, M (s.d.)		3.08(1.10) [1-5.5]
*Neonatal characteristics*		
Gestational age, M (s.d.)		27.22(1.36) [25–31]
Birth weight in grams, M (s.d.)		937(235) [650–1590]
Retinopathy of prematurity, n (%)		1 (3%)
Bronchopulmonary dysplasia, n (%)		15 (45%)
Mild, n (%)		7 (21%)
Moderate, n (%)		5 (15%)
Severe, n (%)		3 (9%)
Intraventricular haemorrhage, n (%)		10 (30%)
Grade I		4 (12%)
Grade II		3 (9)
Grade III		1 (3%)
Grade IV		2 (6%)
Cystic periventricular leukomalacia, n (%)		0
White matter injury grade I, n (%)		4 (12%)
*School-age characteristics*		
Specialized education, n (%)		1 (3%)
Learning difficulties, n (%)		16 (48%)
Language (writing and reading), n (%)		14 (42%)
Math, n (%)		3 (9%)
Other topics, n (%)		3 (9%)
Motor skills and coordination, n (%)		1 (3%)
Global learning difficulties, n (%)		2 (6%)
Memory, n (%)		1 (3%)
Attentional difficulties, n (%)*		17 (51%)
Educational support, n (%)		9 (27%)
Additional time/facilities to enhance attention, n (%)		3 (9%)
Integration support, n (%)		4 (12%)
Adapted program, n (%)		3 (9%)
Therapy, n (%)		14 (42%)
Speech therapy, n (%)		10 (30%)
Psychomotor therapy, n (%)		6 (18%)

* of varying severity, it represents children who met various DSM-V criteria for attention deficit disorder with or without hyperactivity, including one child who was on medication. Note. The school-age characteristics were reported by the parents except for attentional difficulties. These latter were based on a general clinical appreciation including a parent interview, a neuropsychological assessment, observations, and parental questionnaires

### Executive functions and attentional abilities

Overall, the very preterm children in our cohort performed within age-normed standards on each of the neuropsychological tests measuring executive functions and attention. Indeed, most of them performed within the norms (i.e., between 59% and 72%), with only between 13% and 24% of the children performing below the norms (see Table 2 for details).

**Table 2:** Very preterm neuropsychological test scores

	Mean (SD) [range]	Norms	Below the norms (%)	Between the norms (%)	Above the norms (%)
Flanker - inhibition	79.96(20.33) [23.53–100]	/	/	/	/
Flanker - executive functions	57.78(16.01) [10.77–78.46]	/	/	/	/
WISC-V – working memory	103.13(15.23) [65–130]	100 ± 15	12.5	71.9	15.6
CPT - omission	58.34(14.04) [42–90]	45–54 + 5	24.1	58.6	17.2
CPT - commission	52.21(8.04) [37–71]	45–54 + 5	17.2	69	13.8
NEPSY - fluidity	9.94(2.84) [5–16]	10 ± 3	21.9	68.8	9.4

The Flanker is not a standardised test; therefore, there are no norms. However, based on the results of Décaillet et al. (2023), we could say that they performed at an average level for Inhibition but exhibited some difficulties for the executive functions score. For the CPT scores, the lowest scores are the better abilities. *Missing Data*: WISC-V (*n =* 1), CPT (*n* = 3), flanker (*n* = 5)

### Correlations between executive and attentional functions and brain structures

After correction for multiple comparisons, significant negative correlations were found between the cortical thickness of the left ACC and the Inhibition Factor (r(10) = -0.529, *p* = .036) and the Attentiveness Factor (r(10) = -0.466, *p* = .044). In addition, a significant negative correlation was found between the right DLPFC and the Flexibility Factor (r(10) = -0.489, *p* = .042).

Significant negative correlations were observed between the Attentiveness Factor and the FA of the left ACC (r(10) = -0.445, *p* = .016) and the betweenness centrality of the left DLPFC (r(10) = -0.376, *p* = .045). However, these correlations did not survive the correction for multiple comparisons.

No significant correlations were found between the three factors and volume or cortical surface area (see Supplementary Materials).

### Cortical thickness relation with executive and attentional functioning

#### Inhibition factor

**Fig. 1:**
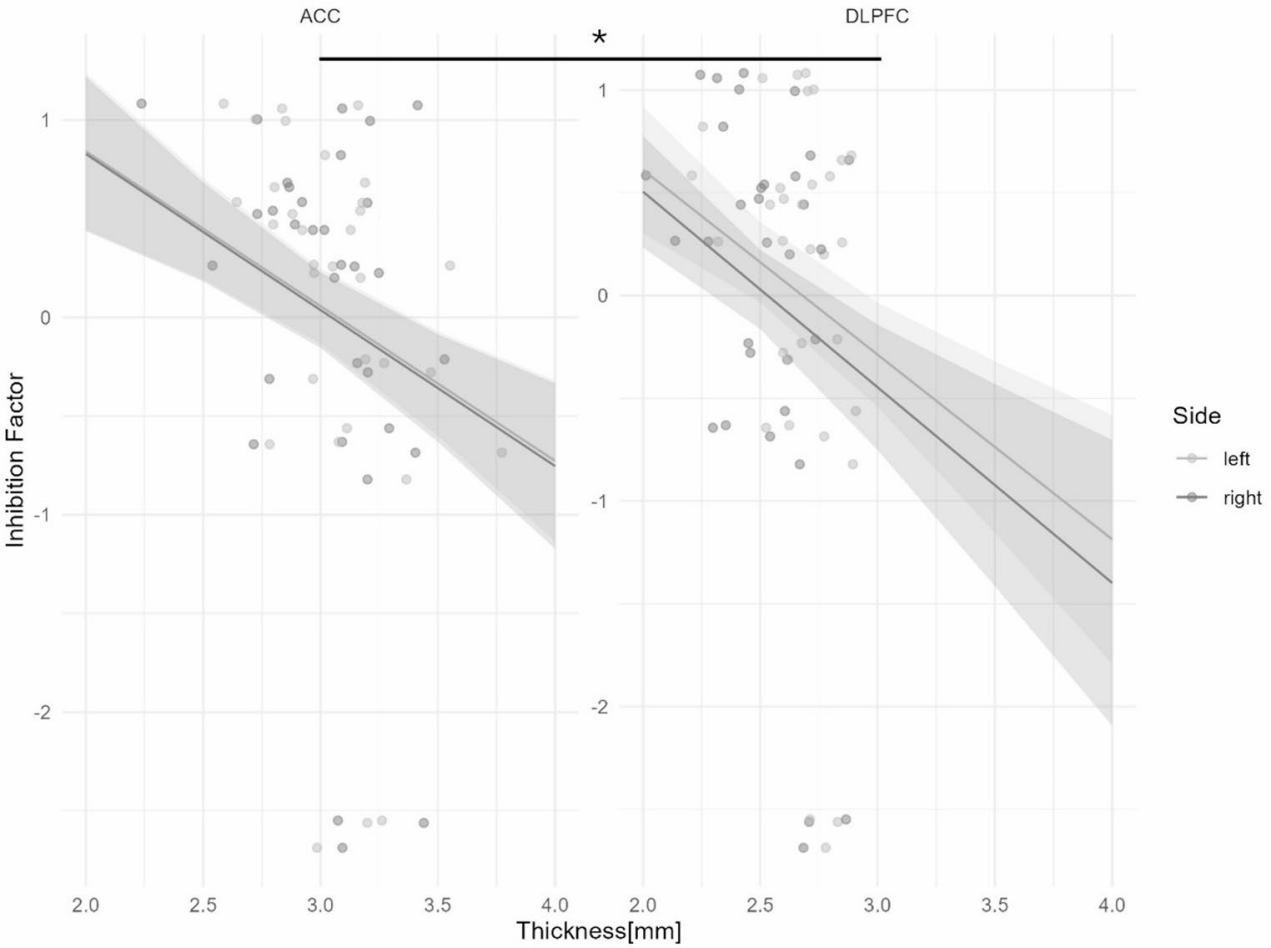
Predicted values of z-scores and actual individual values from the Inhibition Factor according to the cortical thickness depending on hemispheres and brain regions. The analysis revealed a stronger relation for the DLPFC compared to the ACC but no significant influence of the hemisphere. Shaded areas represent the 95% confidence interval. *p < .05

The model with predictors demonstrated a better fit than the null model (χ^2^ = 5.77, df = 5, *p* < .001), and the predictors exhibited no evidence of collinearity (VIF_Thick, Side, Region_ = 1.001, VIF_Sex_ = 1.006). The overall model was statistically significant (F = 4.87, df = 5,122, R^2^ = 13%, *p* < .001) and showed a main effect of sex (β = 0.23, *p* = .009) with higher scores for girls. A significant negative relationship was found between general cortical thickness and the Inhibition Factor (β = -0.78, *p* < .001). In addition, while no effect was found for the hemispheres, the DLPFC demonstrated a stronger negative relationship with the Inhibition Factor (β = -0.11, *p* = .02) (see Fig. 1).

#### Attentiveness factor

**Fig. 2:**
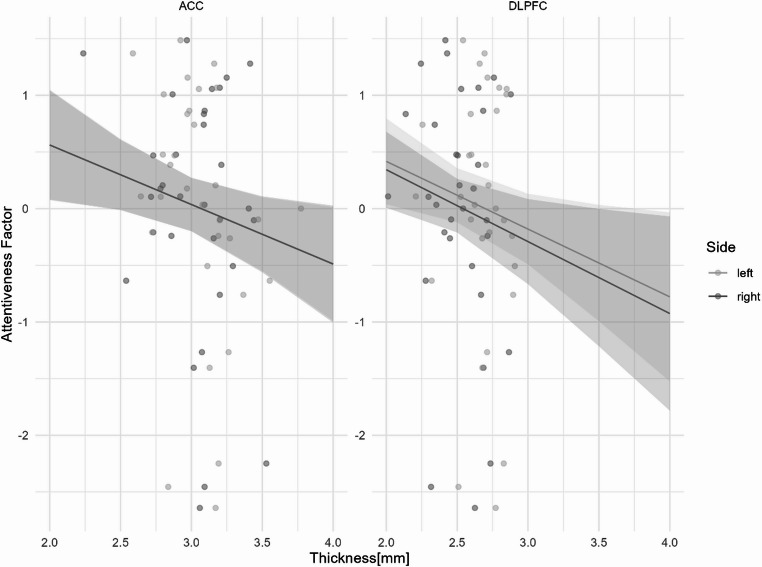
Predicted values of z-scores from the Attentiveness Factor according to the cortical thickness depending on hemispheres and brain regions. No difference between regions and hemispheres were observed. Shaded areas represent the 95% confidence interval

The model with predictors demonstrated a better fit than the null model (χ^2^ = 4.50, df = 5, *p* = .03), and the predictors exhibited no evidence of collinearity (VIF_*Thick, Side, Region*_ = 1.001, VIF_*Sex*_ = 1.006). The overall model was statistically significant (F = 2.501, df = 5,122, R^2^ = 5.56%, *p* = .03), and showed a main effect of sex (β = 0.30, *p* = .005) with higher scores for girls. A significant negative relationship was found between general cortical thickness and Attentiveness Factor (β = -0.53, *p* = .02) but no differences were found for the regions or the hemispheres (see, Fig. 2).

#### Flexibility factor

**Fig. 3:**
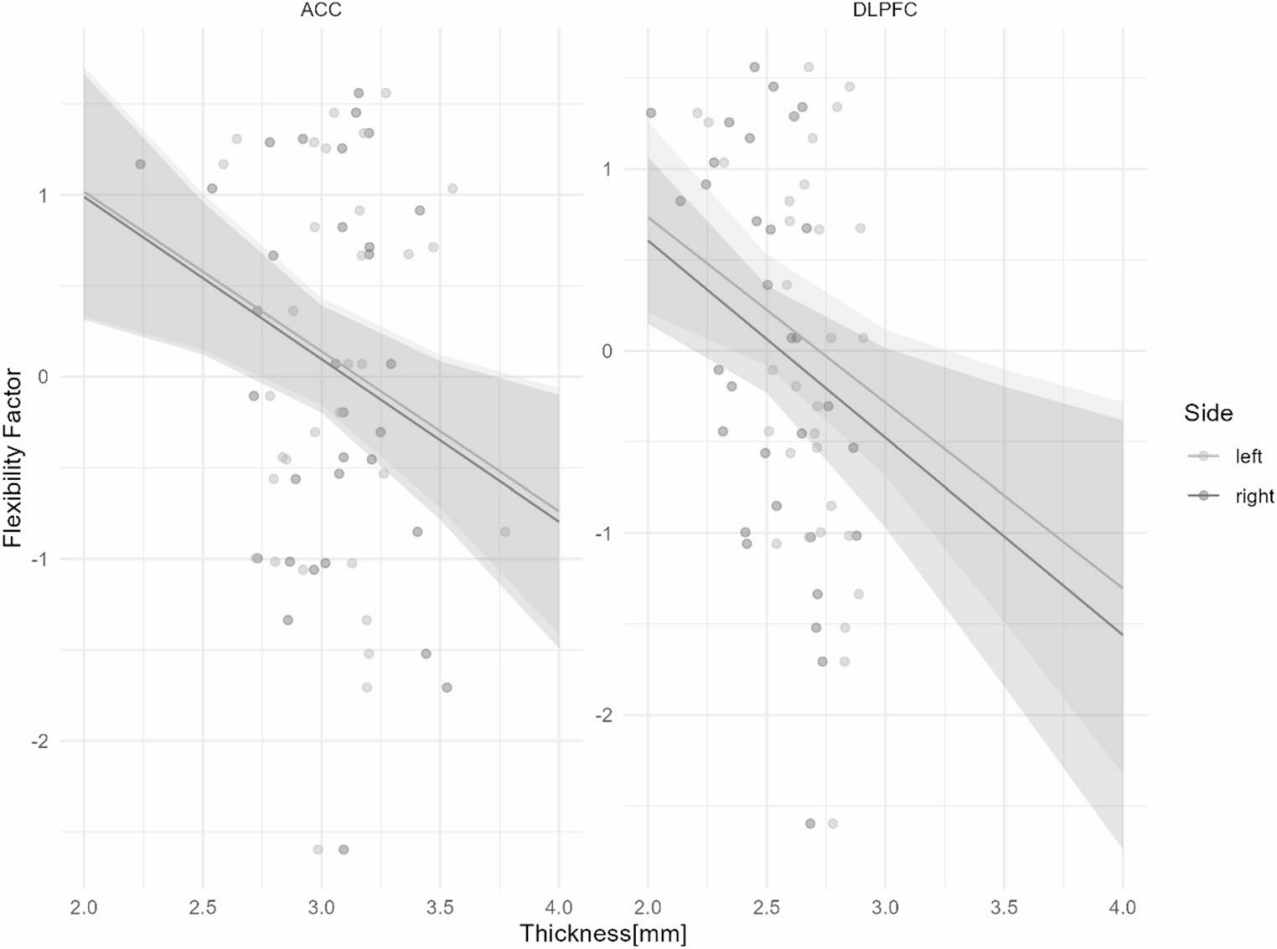
Predicted values of z-scores from the Flexibility Factor according to the cortical thickness depending on hemispheres and brain regions. Note that this model was not statistically significant and no difference between regions and hemispheres was observed. Shaded areas represent the 95% confidence interval

The model with predictors did not demonstrate a better fit than the null model (χ2 = 5.99, df = 4, *p* = .08), and the predictors exhibited no evidence of collinearity. While the overall model was not statistically significant (F = 2.082, df = 4,123, R^2^ = 3.29%, *p* = .087), a significant negative relationship was observed between general cortical thickness and the Flexibility Factor (β = -0.88, *p* = .005). No differences were found for the regions or the hemispheres (see Fig. 3).

### Cortical thickness relationship with executive and attentional functioning in control regions

#### Total brain

The overall model was statistically significant for the Attentiveness Factor (F = 4.98, df = 1.30, R^2^ = 11%, *p* = .03) and the Flexibility Factor (F = 5.74, df = 1.30, R^2^ = 13%, *p* = .02) and significantly better than the null models. A significant negative relationship was found between the general cortical thickness and the Attentiveness Factor (β = -3.68, *p* = .03) and the Flexibility Factor (β = -5.46, *p* = .023). However, regarding the Inhibition Factor, the model with predictors was not better than the null model, not statistically significant and no significant relationship was observed between general cortical thickness and the Inhibition Factor.

### Specific regions

In the fusiform and the inferior temporal cortices, the models with predictors were not better than the null models, the overall models were not statistically significant and showed no significant effect of the general cortical thickness on the Inhibition and Attentiveness Factors. Yet, significant overall models (F_fusiform_ = 3.035, df = 3,60, R^2^ = 8.8%, *p* = .04, and F_inftemp_ = -1.82, df = 2,61, R^2^ = 8.1%, *p* = .03) better than the null models were found for the Flexibility Factor with a significant negative main effect of the general cortical thickness (β_fusiform_ = -2.33, *p* = .006, and β_inftemp_ = -1.82, *p* = .008). In addition, no differences were found between the hemispheres.

The overall models were not statistically significant and no significant relationship between the general cortical thickness and the three factors in the superior temporal, the lateral occipital, and the superior parietal cortices.

## Discussion

The aim of the present study was to comprehensively characterize how executive functions and attentional performances relate to the DLPFC and the ACC structures in very preterm children at school age. Understanding this relationship might help to comprehend their cognitive difficulties and deploy targeted training in the long run. Our finding generally revealed no association between cognitive performance and most of the brain structure’s selected metrics, except for the cortical thickness, where executive functioning and attention capacities were negatively associated with the DLPFC and the ACC cortical thickness.

Notably, half of the very preterm children in our cohort presented functional difficulties with learning difficulties at school or attentional difficulties of varying severity, and 30% of them underwent speech therapy and 18% psychomotricity. The data on the prevalence of these difficulties in the general population in Switzerland is limited. Studies showed that 5% to 7% of children have attention deficit disorder (Bader and Perroud 2012), approximately 5% benefit from an adaptation of their teaching programs (Office fédéral de la statistique 2025), and between 5% and 21% receive speech therapy (DLV 2019). In the United States, 1.7% of the children present learning difficulties (Kesherim 2024). Thus, as generally reported in very preterm children (Pierrat et al. 2021), those in our cohort exhibited slightly more learning difficulties and required more therapeutic interventions. However, these results may be biased by the fact that these are children who are more closely monitored. Nevertheless, the majority demonstrated a cognitive performance that was within the age-standard norms. These results likely reflect improvements in the quality of care and adapted educational and therapeutic support over recent years. They may also be the expression of a selection bias, as severely impaired children who probably were needing extra support were not included in the study. However, it is essential to acknowledge that the present study was conducted in a country where there is access to high-quality care, which is unfortunately not the case in many other countries. Nonetheless, they still demonstrated subtle difficulties in various domains, which can subsequently impact high-order functions, and a large proportion of them experienced functional difficulties (i.e. learning difficulties and therapies) in their daily lives. Therefore, it is imperative to understand the underlying mechanisms of their cognitive performance.

First, we report a potential lack of association between cortical volume and cognitive performance in our cohort of very preterm children. Corroborating past studies, cortical volume seems the least related to cognitive development in very preterm children (De Gamarra-Oca et al. 2024; Murray et al. 2016; Vollmer et al. 2017). That could be partly explained by the fact that cortical volume depends on cortical thickness and surface area (Panizzon et al. 2009). These two metrics could have different developmental trajectories and associations with executive functions and attention.

Secondly, our non-significant result regarding cortical surface area seems to contradict previous findings, which revealed a negative association between cognition and cortical surface area in very preterm children (Mürner-Lavanchy et al. 2018). However, the latter association was found only for the working memory factor, a variable which, in the current study, had only a medium loading in the first factor (i.e., inhibitory abilities). Furthermore, the study above identified this association in the occipital region, in contrast to the regions examined in the present study. In addition, although the correlation did not survive correction for multiple comparisons, we found a negative relation between FA of the left ACC and the Attentiveness Factor. This suggests that greater microstructural organization within the left ACC may be linked to improved attentiveness. While this finding contradicts previous findings showing a positive association between attention and FA, similar negative associations have also been found in other brain regions (Murray et al. 2016). Consequently, our consideration of more general functions as opposed to separate scores, as well as the focus we placed on only two regions mainly involved in executive functions and attention in children, might have affected our non-significant results. Indeed, it is possible that the relationship between the brain and cognition may be more specific or that this relationship may, in fact, lies elsewhere.

Third, to our knowledge, no study has investigated the relation between cognition and betweenness centrality in very preterm or full-term children. Our finding showed that very preterm children may present no association between executive function or attention and the betweenness centrality of the DLPFC and the ACC. Given that betweenness centrality reflects the importance of a region in facilitating information transfer, these findings suggest that the DLPFC and ACC may not play a critical role for the transmission of information related to executive functions and attention at this developmental stage. Instead, these regions may be positioned earlier in the information-processing chain, initiating rather than routing signals through the network (Panikratova et al. 2020).

Finally, we found negative associations between cortical thickness and executive functions and attentional abilities, which is consistent with age-related cortical thinning leading to better cognition (Ducharme et al. 2016; Sowell et al. 2004). Indeed, a synaptic pruning occurs during childhood and adolescence by deleting the weaker synapses and keeping only the most essential synapses to build a more efficient network and an adaptive mature brain (Chechik et al. 1999; Sakai 2020). Nevertheless, this finding corroborates the brain-function relationship of full-term children rather than very preterm children. Indeed, while a negative association was found between working memory, executive function control, and cortical thickness in full-term children, Mürner-Lavanchy et al. (2018) showed a positive association between executive function control and cortical thickness in several regions, with a developmental delay in cortical thickness observed in very preterm children (Mürner-Lavanchy et al. 2014). This might be explained by the fact that most of our very preterm children performed between the norms. However, it should be noted that the developmental trajectory of cortical thickness remains a subject of debate, particularly at this age. The direction of this trajectory is not universally consistent across studies, depending on the type of technique used or the region studied (Walhovd et al. 2016). Moreover, the negative relationship between cognition and cortical thickness varied depending on brain regions and cognitive functions. While the thickness peak appears earlier for somatosensory areas, high-order cortical areas reach it last, reflecting more complex functions (Shaw et al. 2008). In full-term children, Ducharme et al. (2016) describes a cubic developmental trajectory (i.e., an initial increase, then a decline, and finally a stabilisation phase) for the ACC and a quadratic trajectory (i.e., an initial increase then a decline) for the frontal cortex, with a peak thickness around 10.5 years (Shaw et al. 2008). Several studies showed a cortical thinning of the prefrontal cortex during adolescence. This developmental phase is characterized by an increased organization of the prefrontal cortex, which in turn facilitates its capacity to modulate behavioural responses (for reviews see Fleming and McDermott 2024; Tsujimoto 2008). Contrary to previous findings (Sowell et al. 2004), no differences were detected between the hemispheres. However, a stronger negative relationship between cortical thickness and cognition in the DLPFC compared to the ACC was identified only for the factor driven by inhibitory abilities. In addition, to rule out the possibility that the observed association between thinner cortex and better cognitive abilities was only a reflection of a broader association, we examined this relationship throughout the entire brain and in regions unrelated to executive and attentional functions. The present study indeed showed a general negative association between the cortical thickness and the executive and attentional functioning at the level of the entire brain. However, this negative relationship was found to be dependent on the specific regions and cognitive functions investigated, with some of our control regions showing no significant associations or only demonstrating a relationship with the Flexibility Factor. Therefore, while the DLPFC and the ACC are not the only regions associated with executive and attentional abilities, our results showed a certain specificity in this association.

Furthermore, the DLPFC and the ACC are embedded in larger networks that govern executive functions and attentional abilities, specifically the central executive network (i.e., frontoparietal network) and the salience network (i.e., cingulo-opercular network) (Dosenbach et al. 2007; Li et al. 2019). Indeed, while the DLPFC is part of the central executive network which regulates top-down attentional focus, working memory, inhibitory control, and goal-directed behaviour (Mezzacappa 2011), the ACC is a component of the salience network, responsible for conflict monitoring, salience detection, and sustaining attention (Menon and Uddin 2010; Schimmelpfennig et al. 2023). As described in the morphometric and connectivity development, both networks are already present in middle childhood in typically developing children (Engelhardt et al. 2019). However, connectivity between and across theses networks improve during adolescence (Han et al. 2023; Marek et al. 2015; Sherman et al. 2014; Wang et al. 2020) leading to efficient integration and segregation in adulthood, which is related to enhanced executive functions (Fair et al. 2007). Considering these different developmental trajectories, it is essential to explore the relationships between the morphometric and connectivity measures and executive and attentional abilities also during adolescence, when they become mature. Some of our non-significant results may also arise from the developmental stage. Additionally, it would then be beneficial to investigate these associations from a network perspective rather than solely focussing on individual structures.

In addition, we found that girls’ scores at the Inhibition and Attentiveness Factors were higher compared to those of boys. Inconsistent results have previously been found based on the methodology employed to assess inhibition and attentiveness. Greater inhibition capacities have often been reported in girls compared to boys, but recent studies reported a developmental delay in boys (Usai 2023) or no difference in inhibitory control (Sadeghi et al. 2022). Parents reported lower levels of inattentiveness in girls (Kuzmina et al. 2021), although self-reported questionnaires indicated a higher prevalence of the inattentive subtype among teenage girls (Bishry et al. 2018). Furthermore, when measured with the CPT, no differences were observed between girls and boys (Hasson and Fine 2012). Therefore, these mixed results underscore the importance of a multifaceted approach to account for gender biases and no direct conclusion can be derived from our findings.

Several limitations need to be stated. First, while we selected the DLPFC and the ACC based on their well-established roles in executive functions and attention, relevant associations may involve other brain regions, as suggested by prior studies. Future research with larger sample sizes is needed to investigate additional regions and sub-regions, as well as to include more specific cognitive measures. In addition, as mentioned above, the DLPFC and the ACC are not isolated regions, rather they are interconnected with other brain regions. Therefore, it would be worthwhile to also investigate their connections, notably with subcortical brain regions or executive networks. Second, the limited sample size may have underpowered the results. Despite the exploratory factor analysis and the selection of only two specific regions, the absence of significant results for most of the measures may be biased by the relatively modest sample size. Further studies, with a greater number of very preterm children, may be needed to validate the null results. Third, as mentioned above, the present cohort of very preterm children was relatively well-developed and presented only subtle cognitive difficulties. Therefore, including more impaired children might increase brain measures and cognitive performance variability. In addition, future studies may want to compare the brain-function relationship with full-term children or design longitudinal studies to investigate the developmental differences and trajectories. Fourth, the activation maps from Neurosynth were based on adult data.

In conclusion, the present findings provide novel insights into how executive and attentional abilities relate to various brain measures in the DLPFC and the ACC in very preterm children at school age. This study emphasizes the complex relationship between cortical thickness and cognition while questioning the role of the different brain measures according to cognitive regions and functions. A more profound understanding of the difficulties faced by very preterm children and their underlying causes is crucial for comprehending the specificity of their neural and behavioural functioning profiles with the aim at term to improve the support. Consequently, further studies investigating the brain-function relationship, and its implications are necessary for practical and clinical applications.

## Supplementary Information

Below is the link to the electronic supplementary material.

Supplementary Material 1

## Data Availability

The datasets generated during and analysed during the current study are available from the corresponding author on reasonable request.
