# Supplementary material for: Brain structures and their association with executive and attentional abilities in very preterm 8-year-old children
Source: Brain Struct Funct. 2025 Nov 22;230(9):180. doi: 10.1007/s00429-025-03047-8 (PMC12640349; doi:10.1007/s00429-025-03047-8)
Supplement: Supplementary file 1 — Supplementary Material 1 [file 429_2025_3047_MOESM1_ESM.docx]

**Method**

**Factor Analysis**

***Exploratory factor analysis (EFA)***

**
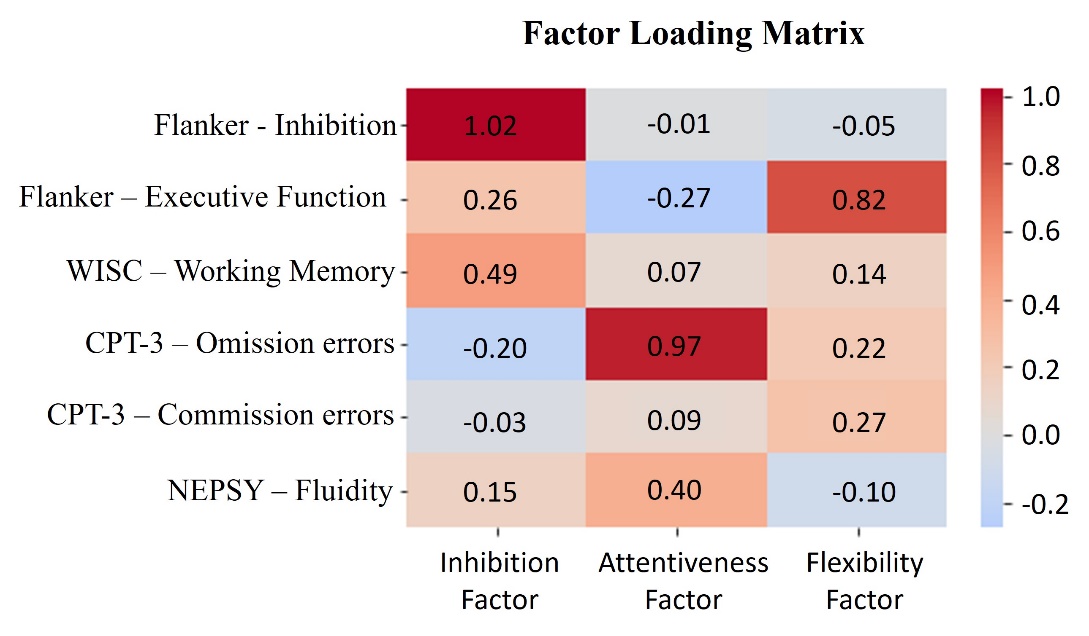
**

**Fig.1** Factor Loadings for the solution with 3 factors. The fraction of variance explained by Inhibition Factor was 0.228, by Attentiveness Factor was 0.190 and by Flexibility Factor was 0.134. The total fraction of variance explained was 0.552.

**Data Analysis**

***Effect of Gender on the Three Factors and Cortical Thickness***

While the simple linear regressions showed a significant effect of gender on Inhibition Factor (β = 0.19, p = 0.041) and Attentiveness Factor (β = 0.28, p = 0.011), no significant effect was found for Flexibility Factor (β = 0.04, p = 0.786) and for thickness (β = 0.05, p = 0.43).


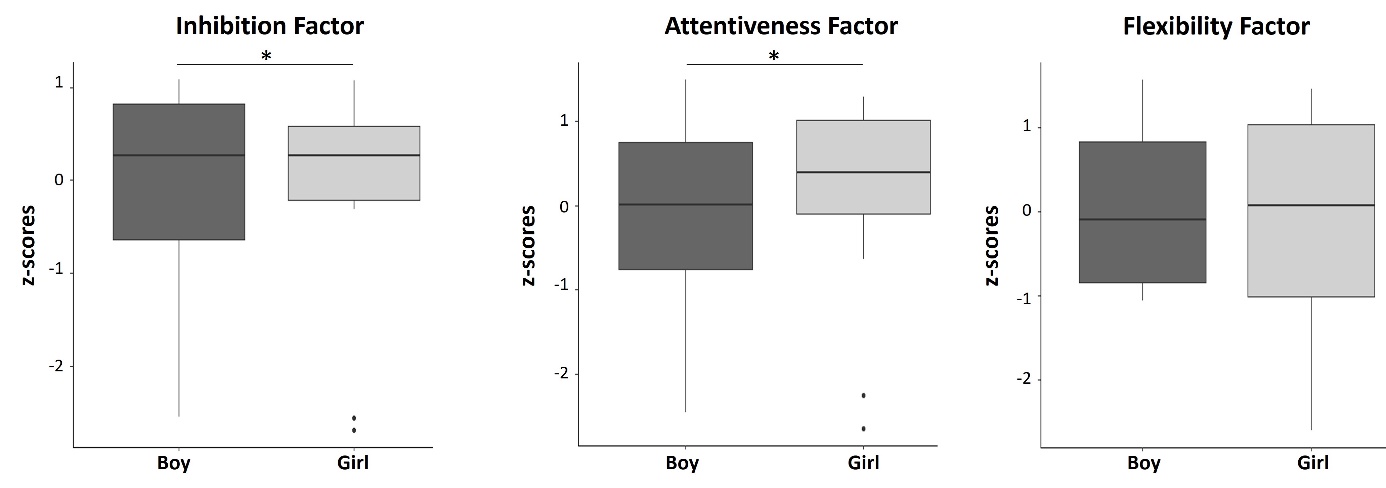


**Fig.2** Differences between boys and girls for the three factors. Note **p*<.05


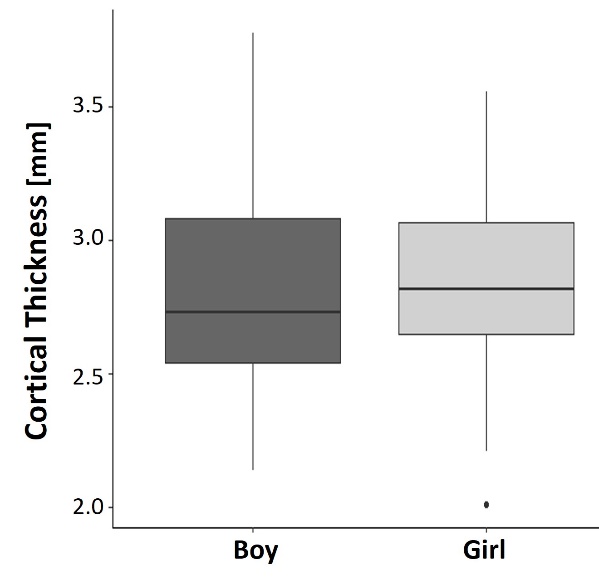


**Fig.3** Difference in cortical thickness between boys and girls

***Effect of Age on the Three Factors and Cortical Thickness***

The simple linear regressions showed no effect of Age on the different factors (i.e., Inhibition Factor (β = -0.11, p = 0.25), Attentiveness Factor (β = -0.04, p = 0.707), Flexibility Factor (β = 0.23, p = 0.154)) and on thickness (β = 0.03, p = 0.47).


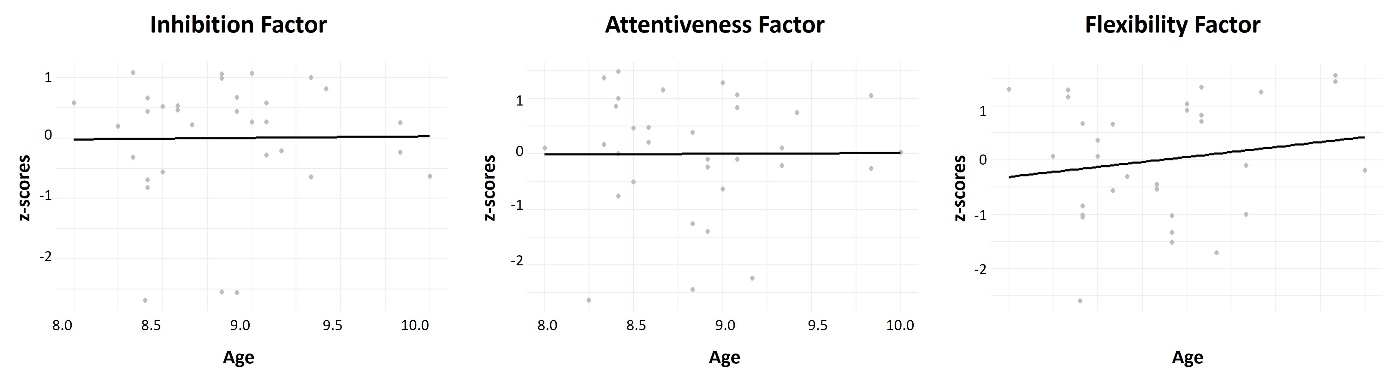


**Fig.4** Relations between age and the three factors


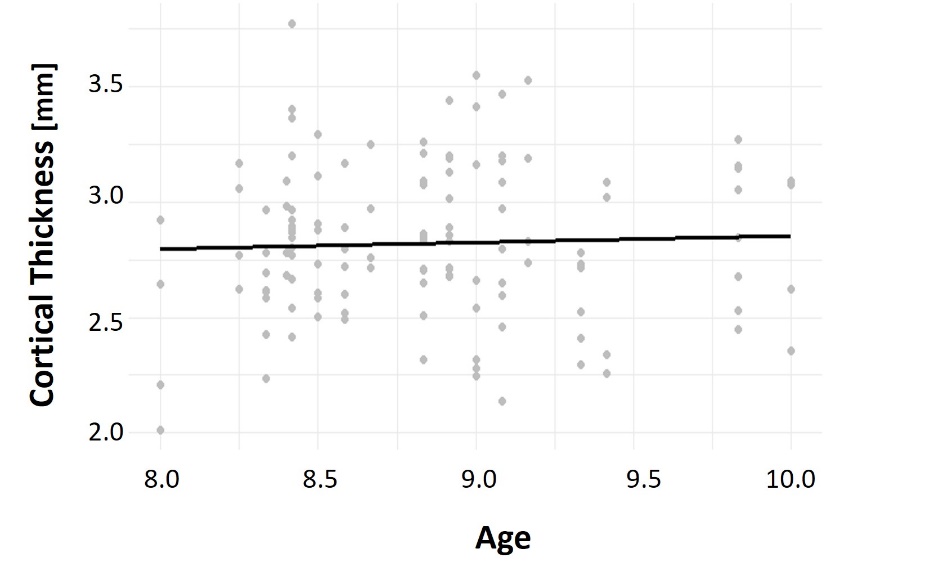


**Fig.5**. Relation between age and cortical thickness

***Effect of SES on the Three Factors and Cortical Thickness***

The simple linear regressions showed no effect of SES on the different factors (i.e., Inhibition Factor (β = -0.063, p = 0.577), Attentiveness Factor (β = -0.069, p = 0.11), Flexibility Factor (β = 0.05, p = 0.382)) and on cortical thickness (β = -0.02, p = 0.24).


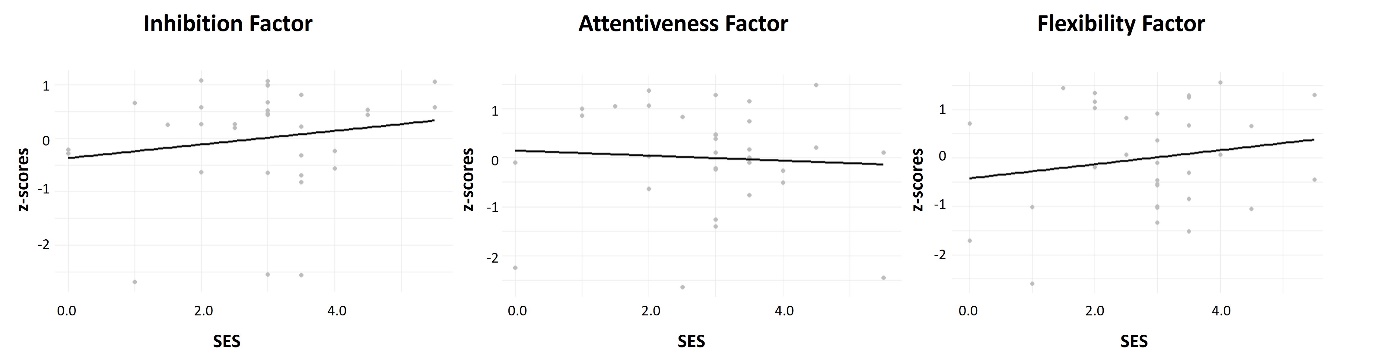


**Fig.6** Relation between SES and the three factors.


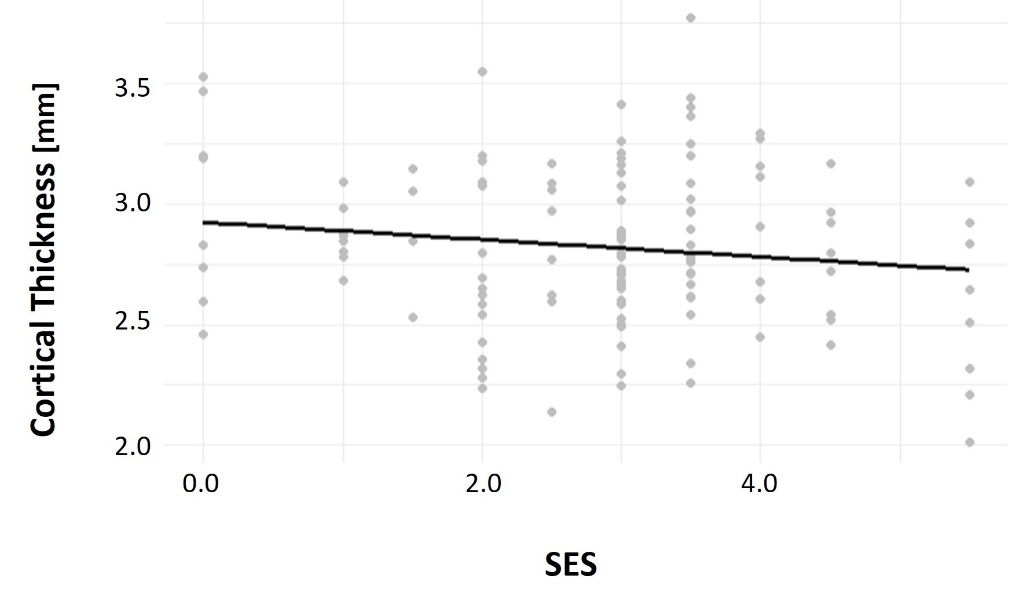


**Fig.7** Relation between SES and cortical thickness

**Results**

**Associations Between Executive and Attentional Functions and Brain Structures**

Theses correlations were adjusted for sex and age and corrected for multiple comparisons.

***Cortical Thickness***

|  | **Inhibition Factor** | **Attentiveness Factor** | **Flexibility Factor** |
| --- | --- | --- | --- |
| **Right DLPFC** | -0.350, p = .149 | -0.268, p = 0.270 | -0.489, p =.042* |
| **Left DLPFC** | -0.256, p = 0.270 | -0.178, p = 0.428 | -0.291, p = 0.250 |
| **Right ACC** | -0.379, p = 0.129 | -0.0.90, p = 0.700 | -0.189, p = 0.428 |
| **Left ACC** | -0.529, p = .036* | -0.466, p = .044* | -0.014, p = 0.943 |

**Table 1.** Correlation coefficients and adjusted p-values for cortical thickness

***Fractional Anisotropy***

|  | **Inhibition Factor** | **Attentiveness Factor** | **Flexibility Factor** |
| --- | --- | --- | --- |
| **Right DLPFC** | 0.028, p = 0.986 | -0.062, p = 0.986 | -0.020, p = 0.986 |
| **Left DLPFC** | 0.116, p = 0.986 | 0.098, p = 0.986 | 0.031, p = 0.986 |
| **Right ACC** | 0.066, p = p = 0.986 | -0.202, p = 0.986 | -0.188, p = 0.986 |
| **Left ACC** | 0.003, p = 0.986 | -0.445, 0.192 | -0.148, p = 0.986 |

**Table 2.** Correlation coefficients and adjusted p-values for fractional anisotropy

***Between Centrality***

|  | **Inhibition Factor** | **Attentiveness Factor** | **Flexibility Factor** |
| --- | --- | --- | --- |
| **Right DLPFC** | -0.235, p = 0.526 | 0.167, p = 0.663 | -0.075, p = 0.970 |
| **Left DLPFC** | -0.260, p = 0.519 | -0.376, p = 0.519 | 0.014, p=0.970 |
| **Right ACC** | -0.178, p = 0.663 | -0.007, p = 0.970 | -0.283, p =0.519 |
| **Left ACC** | 0.041, p = 0.970 | -0.261, p = 0.519 | -0.045, p = 0.970 |

**Table 3.** Correlation coefficients and adjusted p-values for between centrality

***Volume***

|  | **Inhibition Factor** | **Attentiveness Factor** | **Flexibility Factor** |
| --- | --- | --- | --- |
| **Right DLPFC** | -0.179, p = 0.786 | -0.060, p = 0.828 | -0.274, p = 0.786 |
| **Left DLPFC** | -0.003, p = 0.988 | -0.060, p = 0.828 | -0.153, p = 0.786 |
| **Right ACC** | -0.210, p = 0.786 | -0.123, p = 0.786 | -0.102, p = 0.800 |
| **Left ACC** | -0.240, p = 0.786 | -0.147, p = 0.786 | -0.135, p = 0.786 |

**Table 4**. Correlation coefficients and adjusted p-values for volume

***Cortical Surface Area***

|  | **Inhibition Factor** | **Attentiveness Factor** | **Flexibility Factor** |
| --- | --- | --- | --- |
| **Right DLPFC** | -0.055, p = 0.832 | 0.069, p = 0. 888 | -0.075, p = 0. 888 |
| **Left DLPFC** | 0.113, p = 0.832 | -0.078, p = 0. 888 | -0.041, p = 0.832 |
| **Right ACC** | -0.116, p = 0. 888 | -0.112, p = 0. 888 | -0.060, p = 0. 888 |
| **Left ACC** | -0.069, p = 0. 888 | -0.054, p = 0. 888 | -0.226, p = 0. 888 |

**Table 5.** Correlation coefficients and adjusted p-values for cortical surface area
